# Screening of potential target genes for cataract by analyzing mRNA expression profile of mouse *Hsf4*-null lens

**DOI:** 10.1186/s12886-015-0066-3

**Published:** 2015-07-18

**Authors:** Wenjuan Zhao, Wenqing Zhao, Jun Zhao, Dong Wang, Jinghai Li

**Affiliations:** Department of Ophthalmology, Qilu Hospital of Shandong University, Wenhuaxi Road 107, Jinan, Shandong 250012 China; Department of Ophthalmology, Shandong University Affiliated Jinan Central Hospital, Jinan, 250013 China; Department of Neurosurgery, The 5th People’s Hospital of Jinan, Jinan, 250022 China; Health Examination Center, Jinan 2nd People’s Hospital, Jinan, 250001 China; School of Management Science and Engineering, Shandong University of Finance and Economics, Jinan, 250014 China

**Keywords:** Cataract, Differentially expressed gene, Heat shock transcription factor 4, Lens, Module

## Abstract

**Background:**

Hsf4 is closely related to the development of cataract. However, the molecular mechanisms remain unknown. This study aimed to explore the molecular mechanisms that how Hsf4 mutations influence development of lens and thus lead to cataract in mouse.

**Methods:**

The mRNA expression profile of mouse tissue samples from *Hsf4*-null and wile-type lenses was downloaded from Gene Expression Omnibus database. Then the LIMMA package was used to screen differentially expressed genes (DEGs) and DAVID was applied to identify the significantly enriched Gene Ontology (GO) categories for DEGs. Furthermore, the protein-protein interaction (PPI) network of DEGs was constructed using Cytoscape and the key modules were selected from the PPI network based on the MCODE analysis.

**Results:**

A total of 216 DEGs were screened, including 51 up- and 165 down-regulated genes. Meanwhile, nine GO terms were obtained, and DEGs such as *SGK1*, *CRY2* and *REV1* were enriched in response to DNA damage stimulus. Furthermore, 89 DEGs and 99 gene pairs were mapped into the PPI network and Ubc was the hob node. Two key modules, which contained the genes (e.g. *Ubc*, *Egr1*, *Ptgs2*, *Hmox1*, *Cd44*, *Btg2*, *Cyr61* and *Fos*) were related to response to DNA damage stimulus.

**Conclusions:**

The deletion of Hsf4 affects the expression of many genes, such as *Ubc*, *Ptgs2*, *Egr1* and *Fos*. These genes may be involved in the development of cataract and could be used as therapeutic targets for cataract.

## Background

Cataract is a visible opacity in the lens substance which leads to a decrease in vision. The lens is a critical refractive element of the eye which, with the cornea, focuses images of the visual world onto the retina [1]. Previous study has indicated that both the structure and stability of lens crystallins and maintenance of strong cellular homeostatic systems are required for sustaining normal function of lens [2]. Age and genetic component are main factors to influence the development of lens and hence cause cataract [3]. Nowadays, cataract remains the leading cause of blindness in the world, especially in developing countries [4].

Heat shock transcription factor 4 (Hsf4), a member of Hsf family, is the common gene linked to cataractogenesis and it has been regarded as a causative gene for congenital cataract [5]. Hsf4 is expressed exclusively in the ocular lens and acts a key role in the lens formation and differentiation [6]. Besides, Hsf4 regulates DLAD expression and promotes lens de-nucleation [5], and it is involved in the negative regulation of DNA binding activity [7]. Furthermore, Cui et al. have found that Hsf4 promotes DNA damage repair through the regulation of Rad51 expression [8]. It has been also reported that Hsf4 mutations may also be associated with age-related cataract [9] and mutations in the DNA binding domain (A20D, I87V, L115P, R120C and R74H) of Hsf4 cause autosomal dominant cataract [7, 10, 11]. Meanwhile, several studies have reported that mutations located in the other domain of Hsf4 contribute to the autosomal recessive cataract [12–14]. Also, removal of Hsf4 has been found to lead to cataract development in the Hsf4-null (*Hsf4*-/-) mice through reduction of γS-crystallin and Bfsp expression [15].

Gene microarray analysis provides a powerful method for rapid, comprehensive, and quantitative analysis of gene expression profiles of normal/disease states and developmental processes [16]. Thus, the expression levels of thousands of genes can be quantified simultaneously with this technology [17]. Using gene microarray analysis, He et al. [18] have found that Brg1, Hsf4 and Pax6 exert their functions through commonly regulating other genes. However, the molecular mechanisms of Hsf4 are still not fully understood. To further investigate the molecular mechanisms that how mouse Hsf4 (mHsf4) mutations influence lens development and lead to cataract, the mRNA expression profile of *mHsf4*-null mutation and wide-type lens was downloaded from Gene Expression Omnibus (GEO) database deposited by He et al. [18]. Then the differentially expressed genes (DEGs) were identified and used to construct the protein-protein interaction (PPI) network. Furthermore, the significantly enriched functions and important modules were screened and analyzed.

## Methods

### Microarray data and data preprocessing

The mRNA expression profile of GSE22362 [18] was obtained from GEO (available at http://www.ncbi.nlm.nih.gov/geo/) database [19]. The total microarray contains six chips of mouse tissue samples from *Hsf4*-null and wild-type lenses, which were described as a previous study [6]. The expression profile was analyzed by the platform of GPL8321 [Mouse430A_2] Affymetrix Mouse Genome 430A 2.0 Array (Affymetrix, Inc., Santa Clara, CA, USA). Raw data were preprocessed via background correction, quantile normalization and probe summarization using Affy software package [20] of R. Then the probe-level data in CEL files were converted into the mRNA expression values. In the case, if there was more than one probe in a single gene, the average expression values of all probes for a given gene were defined as the mRNA expression value. Meanwhile, when several mRNAs were mapped by one probe, this probe was thought to lack specificity, and was removed from the analysis.

### Screening of DEGs

The wild-type samples were classed as the controls ant the normalized data were analyzed using LIMMA (Linear Models for Microarray Data, available at http://www.bioconductor.org/packages/release/bioc/html/limma.html, V 3.22.1) package [21]. Then the *p*-value was adjusted into FDR (false discovery rate) [22] by Bonferroni method [23] in multtest package. The mRNAs with the cutoff criteria of |log_2_fold change (FC)| >1 and FDR <0.05 were considered to be DEGs. Furthermore, to explore whether the mRNAs were samples-specific, Pheatmap package (available at http://cran.r-project.org/web/packages/pheatmap/index.html, V 0.7.7) [24] in R was used to perform hierarchical clustering by comparing the value of each mRNA in six samples.

### Functional enrichment analysis

The Database for Annotation, Visualization and Integrated Discovery (DAVID) [25] is the most common tool to analysis the functional enrichment of genes. To identify the functions of DEGs, the DAVID was used to identify the significantly enriched GO (Gene Ontology) categories. The *p*-value <0.05 was selected as cutoff criterion.

### Construction of PPI network

The Search Tool for the Retrieval of Interacting Genes (STRING, available at http://www.string-db.org/) database is a useful tool that provides lots of experimental and predicted information of proteins [26, 27]. In order to research the relationship between genes, the DEGs were scanned by the STRING and the PPI pairs were selected with the cutoff criterion of combined score >0.4. Then the PPI network was visualized using Cytoscape (available at http://www.cytoscape.org/) [28].

### Screening of modules

Proteins encoded by genes in the same module may perform the same or similar functions. To further explore the functions of proteins, the network modules were obtained from the PPI network based on the MCODE analysis [29]. Default parameters (Degree Cutoff: 2, K-Core: 2) were set as the threshold for modules screening.

## Results

### DEGs screening

**Fig. 1:**
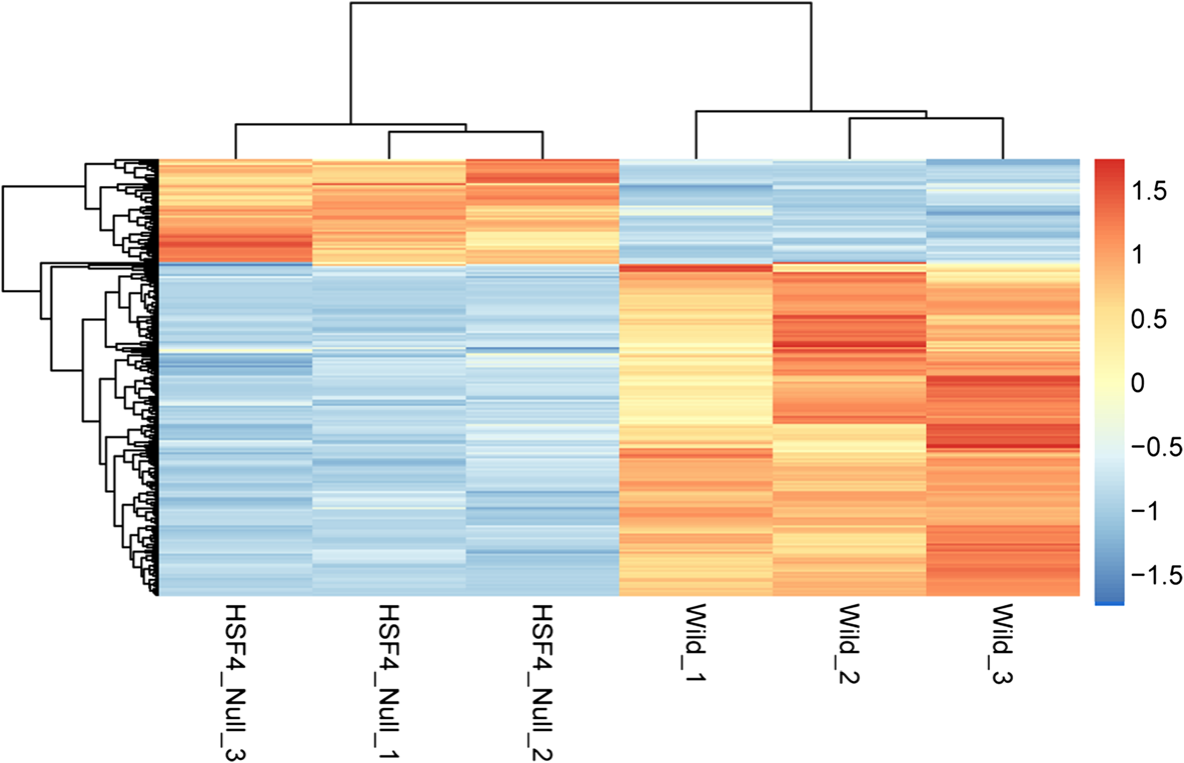
The hierarchical clustering diagram of mRNA expression. Each column corresponds to a single microarray whereas each row indicates expression profile of a single gene. Red and blue stand for high and low values in the mRNA expression, respectively. The expression value from low to high was showed by gradient of blue to red

A set of 216 DEGs were identified in the *Hsf4*-null samples compared with wide-type samples, including 51 up- and 165 down-regulated DEGs. Besides, the hierarchical clustering analysis indicated that the DEGs in *Hsf4*-null samples were distinguished from that in wide-type controls (Fig. 1).

### Functional enrichment analysis

**Fig. 2:**
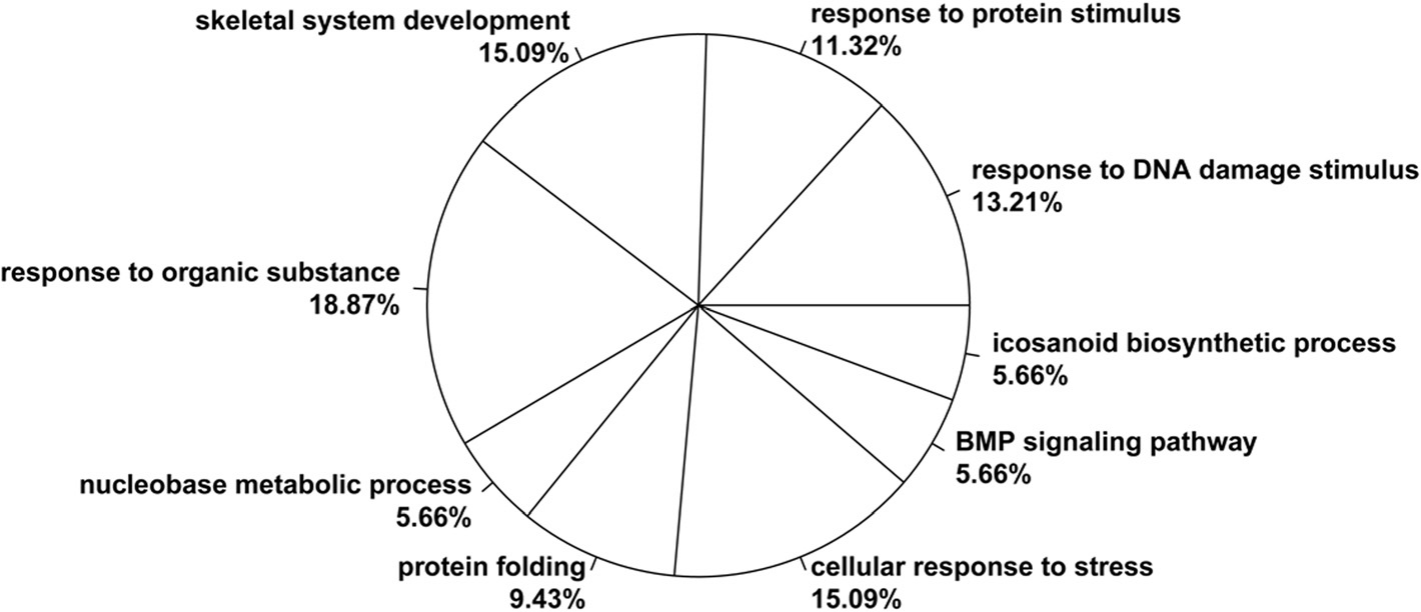
The enriched gene ontology biological processes of differentially expressed genes

In total, nine GO biological processes were obtained (Fig. 2). Among these functions, DEGs such as *BTG2*, *HMOX1* and *REV1* were significantly enriched in response to DNA damage stimulus (*p* = 5.01E-05); DEGs such as *FOS*, *EGR1* and *MSX1* were distinctly enriched in response to protein stimulus (*p* = 1.16E-04); DEGs such as *PTGS2*, *ACVR2A* and *ALOX15* were markedly enriched in skeletal system development (*p* = 9.74E-04) (Table 1).

**Table 1:** The enriched GO terms of differentially expressed genes

Term	Count	*p*-value	Genes
GO:0006974 ~ response to DNA damage stimulus	7	5.01E-05	*SGK1*, *CRY2*, *REV1*, *TIMELESS*, *BTG2*, *DTL*, *HMOX1*
GO:0051789 ~ response to protein stimulus	6	1.16E-04	*FOS*,*EGR1*, *MSX1*, *HSPA4L*, *FAS*, *CYR61*
GO:0001501 ~ skeletal system development	8	9.74E-04	*PTGS2*,*ACVR2A*, *ALOX15*, *MSX1*, *UBC*, *COL1A1*, *BMPR1A*, *IDUA*
GO:0010033 ~ response to organic substance	10	1.79E-03	*FOS*, *EGR1*,*SGK1*, *MSX1*, *PYGM*, *HMOX1*, *HSPA4L*, *COL1A1*, *FAS*, *CYR61*
GO:0009112 ~ nucleobase metabolic process	3	4.84E-03	*UMPS*, *UOX*, *PPAT*
GO:0006457 ~ protein folding	5	5.30E-03	*HSPH1*, *HSPA4L*, *DNAJB1*, *DNAJB4*, *DNAJA4*
GO:0033554 ~ cellular response to stress	8	6.81E-03	*SGK1*, *CRY2*, *REV1*, *TIMELESS*, *BTG2*, *DTL*, *HMOX1*, *MAPK10*
GO:0030509 ~ BMP signaling pathway	3	7.67E-03	*ACVR2A*, *MSX1*, *BMPR1A*
GO:0046456 ~ icosanoid biosynthetic process	3	9.65E-03	*ALOX15*, *PTGS2*, *ALOX12*

*GO* gene ontology

### PPI network construction

**Fig. 3:**
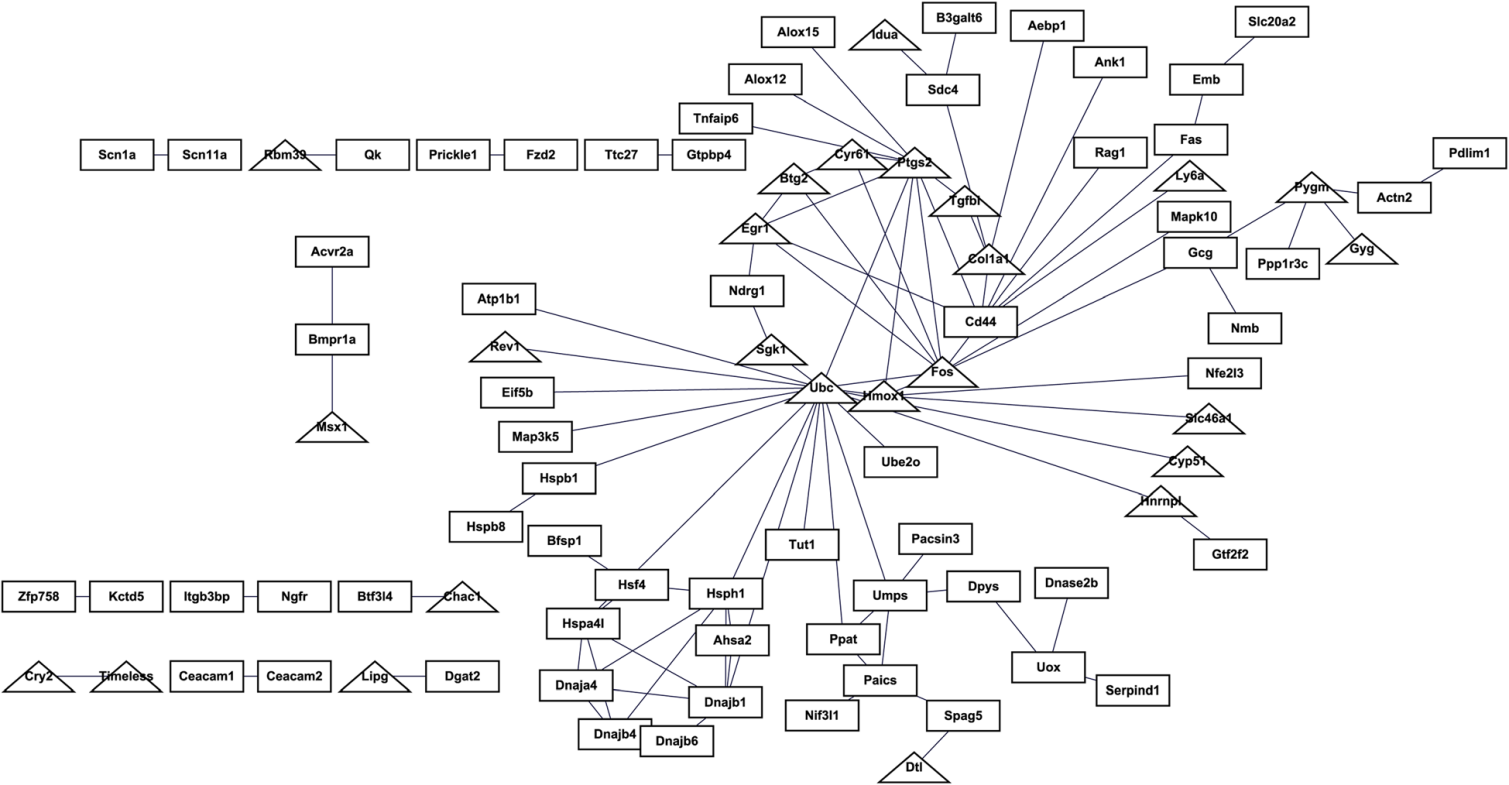
The protein-protein interaction network of differentially expressed genes (DEGs). The triangles and rectangles indicate up- and down-regulated DEGs, respectively

Among the 261 DGEs, 99 gene pairs were identified with the combined score >0.4 and 89 DEGs (25 up- and 64 down-regulated) were mapped into the PPI network (Fig. 3). Several nodes had higher connectivity degrees in the PPI network, such as Ubc (degree = 18), Ptgs2 (degree = 10), Fos (degree = 9), Cd44 (degree =8), Hsph1 (degree = 6), and Gnajb1 (degree = 6) (Table 2).

**Table 2:** Differentially expressed genes with the top 10 % connectivity degree in the protein-protein interaction network

Node	Degree	Node	Degree
Ubc	18	Dnajb1	6
Ptgs2	10	Hmox1	5
Fos	9	Egr1	5
Cd44	8	Hspa4l	5
Hsph1	6	Umps	5

### Screening of modules

**Fig. 4:**
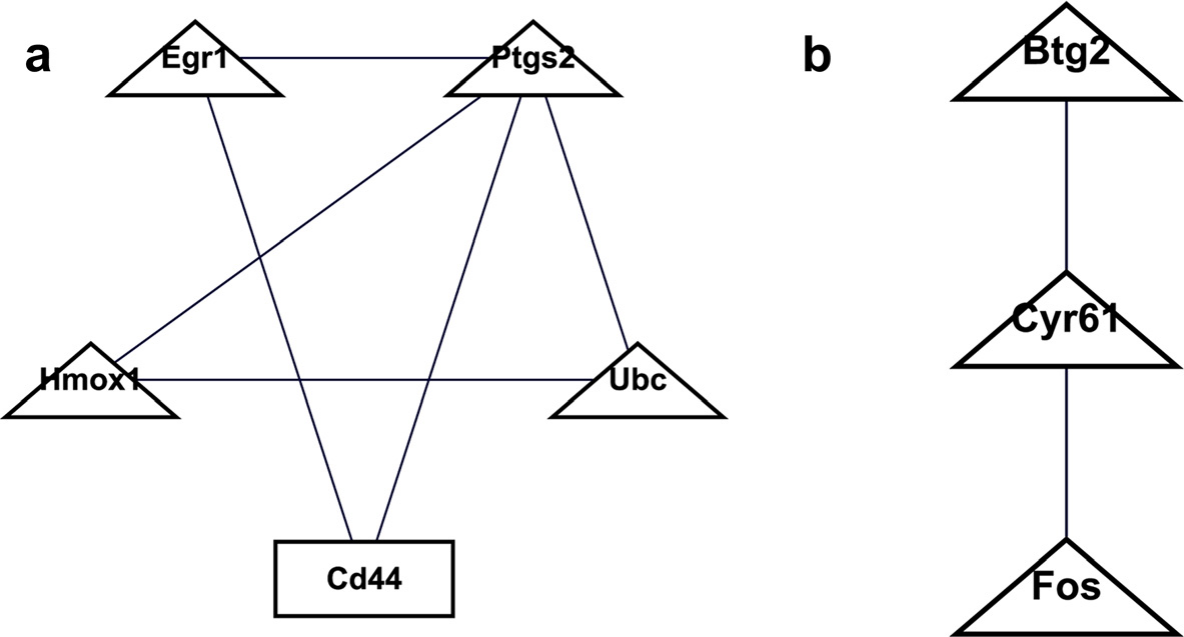
Modules for differentially expressed genes related to response to DNA damage stimulus. **a** module 1; **b** module 2. The triangles and rectangles indicate up- and down-regulated DEGs, respectively

The GO enrichment analysis indicated that response to DNA damage stimulus was the most significant function. To further understand the relationships between DNA damage and cataract, MCODE was used to identify the functional modules of the genes that were related to DNA damage. As a result, two modules were screened. Module one contained Ubc, Egr1, Ptgs2, Hmox1 and Cd44 and module two contained Btg2, Cyr61 and Fos (Fig. 4).

## Discussion

Cataract is the opacification of the eye lens, and is the leading cause of blindness worldwide [30]. Cataractogenesis has multiple causes and is often associated with an abnormality of the lens microarchitecture [31]. Hsf4 is prominently expressed in the lens compared with in other tissues and closely related to the development of cataract [5, 32]. In the present study, we aimed to extend our understanding of the influence of lens development caused by *Hsf4* mutations. Results revealed that expression levels of 216 genes were altered in *mHsf4*-null lens compared with wide-type controls. Functional enrichment results showed that response to DNA damage stimulus was the most significant function in *mHsf4*-null lens. In addition, we identified two key modules correlated with response to DNA damage stimulus from the PPI network.

Cataracts may be caused primarily by the DNA damage, such as oxidized purines [33] and DNA single strand breaks [34]. Study has found that oxidative DNA damage is significantly high in the lens epithelial cells (LECs) of cataract patients [35, 36]. Besides, loss-of-function mutations in TBC1D20 cause cataracts in blind sterile mice [37]. The DNA damage response (DDR) is a signal transduction pathway that senses DNA damage and sets a response to protect the cell and moderate the threat to the organism [38, 39]. Mice with the knock-down of HSF4 have cataract because of an increased proliferation of LECs in the lens as well as an abnormal lens fiber cell differentiation [8]. Two modules which were closely related to DDR, were isolated from the PPI network. Ubc (ubiquitin C/polyubiquitin-C), the module-related gene, was also showed to be the hub node in the PPI network. Ubc is one of the sources of ubiquitin during cell proliferation and stress that cannot be compensated by other ubiquitin [40]. Ubiquitin is a normal component in the lens and a ubiquitin-dependent proteolytic system exists in lens [41], which consists of fiber cells that differentiate from epithelial cells and undergo programmed organelle degradation during terminal differentiation [42]. Furthermore, ubiquitination is a reversible post-translational modification of cellular proteins and is considered to play key roles in the regulation of varieties of cellular processes, such as protein degradation, cell-cycle regulation, DNA repair, apoptosis and signal transduction [43]. Moreover, the ubiquitin proteasome system is found to be essential to cell proliferation of the lens epithelium and required for differentiation of lens fiber cells in zebrafish [44]. The overexpression of ubiquitin affects ubiquitin proteasome system and thus disorders cell proliferation and differentiation of lens. Thus our results suggested that the lack of Hsf4 up-regulated the expression of Ubc, which might be involved in the development of cataract by regulating the cellular processes of lens.

Ptgs2 (prostaglandin-endoperoxide synthase 2) is also known as cyclooxygenase-2 or COX-2, which is involved in the conversion of arachidonic acid prostaglandin H2. In human fibroblasts, Ptgs2 has been showed to interact with Cav1 (Caveolin 1) [45], which is the main component of the caveolae plasma membranes. Caveolae is cholesterol-rich lipid rafts that are likely to play important roles in lens [46]. What is more, Cav1 was found to participate in repair of DNA damage through regulating the important molecules involved in maintaining genomic integrity [47]. Besides, redundant Cav1 has been reported to play a role in age-dependent hyporesponsiveness to growth factors *in vitro* and may act as an indicator of wound-healing capacity in aged human corneal epithelium [48]. Therefore, Ptgs2 plays a role in the response to DNA damage and may be related to the repair of DNA damage in lens through the interaction with Cav1.

Egr1 (early growth response 1) belongs to EGR family of zinc finger proteins and functions as a transcriptional regulator. It has been reported that the mRNA expression of Egr1 can be used as a marker for the direction of mammalian ocular growth [49]. In addition, Fos (FBJ murine osteosarcoma viral oncogene homolog), also named c-FOS, can be induced by a variety of extracellular stimuli [50] and interact with Jun (jun proto-oncogene, c-JUN) to form the transcription factor AP-1 (activating protein 1) [51], which regulates cell adaptation to environmental changes [52]. Furthermore, Fos and Jun are differentially regulated during terminal differentiation of lens fiber cells [53]. Thus, Egr1 and Fos may be involved in the cell cycle and apoptosis of lens.

However, there were some limitations in this study. For example, there were no experiments to confirm our predictions. The number of samples were also small. Considering these issues, the experimental studies will be subsequently conducted later using more samples.

## Conclusions

In conclusion, the deficiency of Hsf4 affect the expression of a set of genes, especially *Ubc*, *Ptgs2*, *Egr1* and *Fos*, which are closely related to the response to DNA damage stimulus. These genes may be participated in the development of cataract by influencing the cellular activities of lens and could be used as therapeutic targets for cataract if they were validated by the further experiments which would be conducted later.

## Abbreviations

DEGs: Differentially expressed genes
GO: Gene ontology
PPI: Protein-protein interaction
Hsf4: Heat shock transcription factor 4
Egr1: Early growth response 1
